# Investigation of Gel Properties of Novel Cryo-Clay-Silica Polymer Networks

**DOI:** 10.3390/gels6020011

**Published:** 2020-03-30

**Authors:** Patrik Berg, Carsten Dieter Prowald, Dirk Kuckling

**Affiliations:** Paderborn University, Faculty of Science, Department of Chemistry, Warburger Str. 100, 33098 Paderborn, Germany; patrik.berg@uni-paderborn.de (P.B.); proc@mail.uni-paderborn.de (C.D.P.)

**Keywords:** cryo, clay, silica, gel, physical properties, post modification

## Abstract

Several methods to increase the mechanical and swelling properties of Poly(*N*-isopropylacrylamide) gels are known. In this study different methods were combined to systematically alter the gel properties. The combination of nanocomposite and cryo gels as well as silica post modification was used to modulate the gel strength. This new cryo-clay-silica gel based on *N*-isopropylacrylamide was investigated in respect to degree of swelling, kinetic of thermo responsive behavior and tensile strength. Here, the properties of new cryo-clay-silica gel were compared with properties of clay-, silica-clay and cryo-clay gels.

## 1. Introduction

Polymer gels are an important field in scientific research due to their adjustable properties and, hence, a variety of applications were known for these type of polymers [1]. The degree of swelling as well the mechanical properties are important parameters. By crosslinking linear polymer chains the tensile properties were limited with a counterbalance between elongation and strain [2,3]. Since the 1960s, it is known to improve polymer network properties by combining polymers with nanocomposites [4,5]. Recently, much attention is paid to this research field [6], for example, by reaction of hydrogels with silica. The polymer silica gel hybrids showed an increase in stiffness due to the generation of a second type of network [7]. Oppositely, treating a chemically cross-linked gel with silica nanoparticles adsorption of the polymer chains onto the nanoparticles forms a second type of cross-links (physical) [7]. This combination could successfully be transferred to the preparation of polymer-clay composites. These composites were also used to improve the physical properties of gels. The combination with artificial clay minerals resulted in gels with high strain and elongation [8,9]. In a certain range of gel composition other physical properties like thermo responsive behavior was not influenced [10,11]. For polymer-clay gels a variety of different synthesis methods were investigated. For example, it was observed that the properties of gels could be influenced after synthesis by thermal post treatment [12]. By post treatment with tetraethyl orthosilicate (TEOS), the mechanical properties like stiffness were increased, while decreasing the elasticity in comparison to unmodified clay gels [13]. By treatment of an existing polymer-clay network with TEOS and following in situ hydrolysis and polycondensation the formation of a second networks occurs. The silica did not form nanoparticles, but it cross-links the clay sheets of the polymer-clay composite. With this second cross-linking the stiffness was reinforced due to increasing cross-linking density [13]. In an alternative preparation method, so-called cryo-clay gels could be prepared at low temperature. By this process polymer gel properties could be influenced via cooling rate and freeze time of monomer solution [14]. This type of preparation method could also successfully be transferred to polymer-clay composites to influence the elasticity as well as the tensile strength in dependence of the clay content and cooling time [15].

In the literature, different routes to synthesize hydrogels are described that enable a definition of gel properties during the initial synthesis. The combination of polymers with nanocomposites and the successful amplification with silica as well as synthesis at low temperature to influence gel properties encouraged us to study the combination of all different synthesis routes within one polymer gel. Cryogels were often used for biomedical application because of their porous structure and mechanical stability [16]. The use of cryogel nanocomposites is an ongoing field of biomedical research [17,18]. Here, a study dealing with the investigation of gel properties of new cryo-clay-silica gel, and a comparison with the common counterparts is presented. With such kind of gels, an additional route for adjusting cryogel properties was facilitated. 

## 2. Results and Discussion

**Figure 1:**
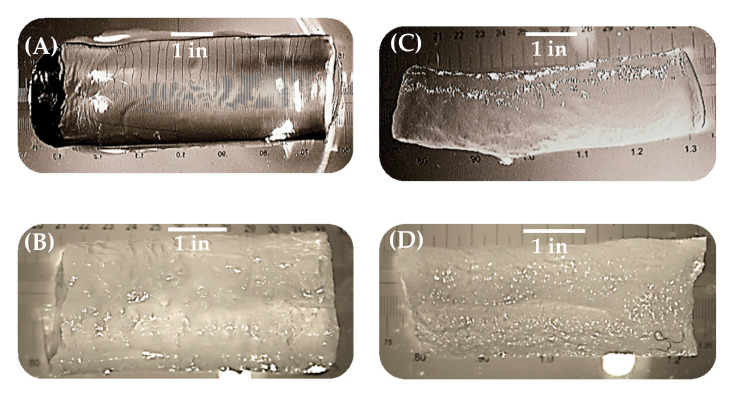
Light microscope images (scaling bar represents 1 inch) of prepared gel samples, completely swollen in water for illustration of different turbidity levels due to change of preparation method; (**A**) image of polymer-clay (NC-N), (**B**) polymer-clay-silica (NC-NT), (**C**) polymer-cryo-clay (NC-C) and (**D**) polymer-cryo-clay-silica (NC-CT) composite gel.

In order to systematically study the influence of the preparation conditions different Poly(*N*-isopropylacrylamide) (PNIPAAm) hydrogels like polymer-clay (NC-N), polymer-clay-silica (NC-NT), polymer-cryo-clay (NC-C) and polymer-cryo-clay-silica (NC-CT) composite gels were prepared. By using thermogravimetry (TGA) up to 700 °C the clay content as well the silica content of each samples was determined [13]. It was observed that 40 wt% (NC-N) and 39 wt% (NC-C) residues remained. This result represents the initial monomer/clay ratio during the synthesis. After silica modification, equally prepared samples show 47 wt% (NC-NT) and 49 wt% (NC-CT). By calculating the difference of TGA results, the silica content was 7 wt% (NC-CT) and 10 wt% (NC-CT), respectively. These results are in accordance with the preparation procedure [13]. First an optical analysis of gel samples was done by qualitative comparison of turbidity of the prepared samples. In the fully swollen state, the prepared polymer-clay gel (NC-N) was transparent (Figure 1A). By changing of the preparation method, the gels became cloudy (Figure 1B–D). For the polymer-clay-silica (NC-NT) gels the refractive index was slightly increased, while the gel lost transparency (Figure 1B). For polymer-cryo-clay gels (NC-C) this effect was increased, and the gels became cloudier (Figure 1C). For the last modification, the combination of all types of synthesis methods to prepare the polymer-cryo-clay-silica (NC-CT) gels was observed and it was seen that the effect was further increased (Figure 1D).

An important property of polymer gels is the degree of swelling. To be comparable, the swelling degrees of NC-N, NC-NT, NC-C and NC-CT were calculated at 25 °C in comparison to the dried state. The results are summarized in Table 1. Simple NC-N gels showed the highest swelling while cryo preparation decreased the swelling properties slightly. The preparation of polymer composites with the silica post modification decreased the volume degree of swelling in comparison to NC-N gels dramatically. The volume degree of swelling for new NC-CT gels was the smallest of all samples. Due to the porous structure of NC-C, the penetration of TEOS is pronounced and a denser silica network can be formed preventing the size change of the composite hydrogel.

**Figure 2:**
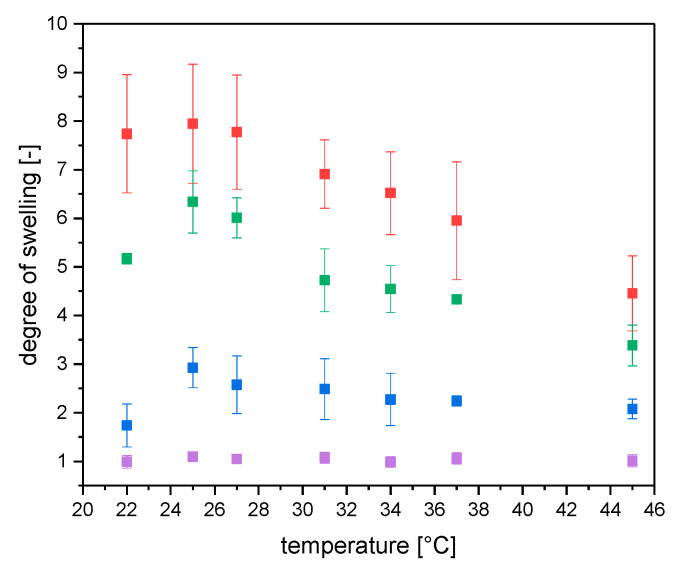
Temperature depending volume degree of swelling of different polymer-clay composites. Red—NC-N; blue—NC-NT; green—NC-C; purple—NC-CT. Error bars describe standard deviation of a minimum of 3 samples; if there no error bar than the error is smaller than the datapoints themselves.

The use of *N*-isopropylacrylamide (NIPAAm) monomer introduced a thermo responsive behavior to the composite gels. For determination of the critical phase transition temperature (CPTT), the influence of temperature on volume degree of swelling was investigated. It was expected that with heating the samples above the CPTT a sharp phase transition could be observed. In Figure 2 it can be seen that the onset of CPTT was shifted to lower temperatures (maximum at 25 °C) compared to simple PNIPAAm [19]. The CPTT behavior was changed from almost discontinuous decrease of degree of swelling to shallow gradual decrease. The shift and decrease of CPTT response were observed earlier as well. Several papers discussed similar behavior for CPTT of nanocomposite gels [11,20]. Further studies showed that this effect is depending on clay content of nanocomposite gels [11]. In Figure 2 this effect was observed for NC-N, NC-NT and NC-C gels. For new NC-CT gels almost no temperature induced effect was observed resulting in a small degree of swelling. An increase of temperature during these measurements above 45 °C was not suitable. In contrast to NC-N and NC-C gels, the silica post modified gels started floating on the solvent surface, by which the volume determination was impossible.

Haraguchi et al. showed that a high clay content decreased the degree of swelling at 20 °C from 6 (NC-1) to 3 (NC-20) [11]. Here, different preparation methods were compared keeping the clay content constant. Two different groups of gels can be recognized, without TEOS (NC-N/NC-C) and with TEOS treatment (NC-NT/NC-CT) showing similar behavior of volume degree of swelling. The degree of swelling was decreased by switching preparation method to a lower temperature (smaller influence) as well as the post modification with silica (stronger influence). The silica framework makes the gels stiffer and prevents the network to swell to the same extent as the unmodified gels. 

**Figure 3:**
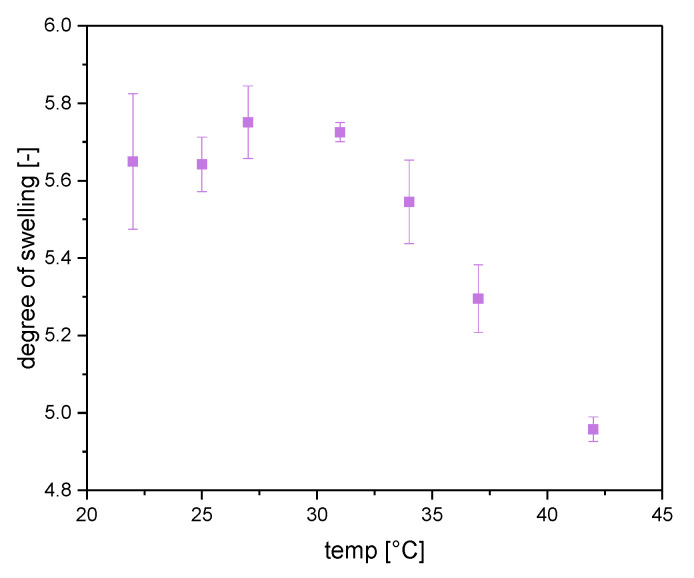
Temperature depending mass degree of swelling of NC-CT gels. Error bars describes standard deviation of 3 differently prepared samples.

Clay nanocomposites inside polymers are known to increase acceptance of tensile strength to synthesized gels [13]. Here, common preparation methods are compared with new cryo-clay-silica gel. Figure 2 showed that there was a high difference between the volume degree of swelling of different samples. The prepared NC-CT gels showed the smallest swelling degree of all samples. Since it was difficult to adjust the volume of gels to a comparable value, the mass depending degree of swelling was used for analysis of tensile strength. Therefore, a fixed amount of water was added to the dried samples corresponding to the desired degree of swelling. At last, the samples were stored to reach equilibration inside a closed vessel for several hours. To obtain comparable values all samples were swollen with water to 300 wt%. This value corresponds to a value below maximum swelling of NC-CT gel, which were the restricting gels with smallest degree of swelling (compare Table 1 and Figure 3). It has to be pointed out that according to Figure 2a volume degree of swelling of approximately 1 for NC-CT, independent on solvent temperature, was determined. In contrast, the mass averaged degree of swelling showed a different behavior. A CPTT for NC-CT of around 31 °C was observed. The CPTT behavior (Figure 3) was in accordance with the observed floating of NC-CT samples at a temperature of higher than 45 °C, due to the decrease of sample density.

**Figure 4:**
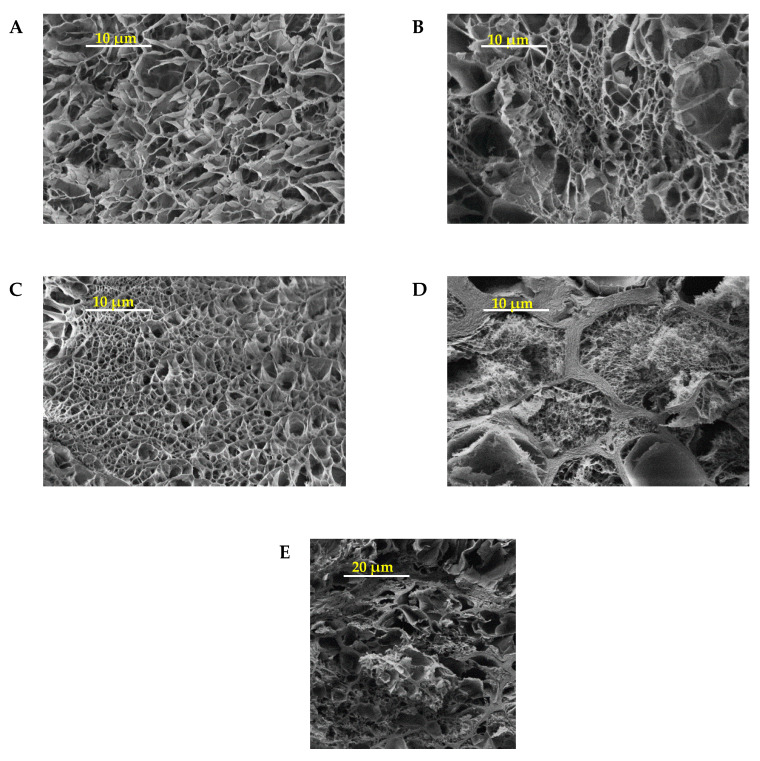
SEM images with 2000-fold magnification of (**A**) NC-N, (**B**) NC-NT, (**C**) NC-C and (**D**) NC-CT gels. Scaling bar represents 10 µm. (**E**) 500-fold magnification of NC-CT gels. Scaling bar represents 20 µm.

The difference between volume and mass degree of swelling of NC-CT gels can be explained with the density of polymer networks. Clay gels have a density of around 1.5 g/cm³ depending on clay amount [21]. In contrast the dried NC-CT gels had lower density of 0.6 (±0.1) g/cm³, which is in the typical range for silica modified aerogels [22]. Due to the cryogenic structure of the gel TEOS forms a more dense porous silica structure maintaining a constant volume. This more porous NC-CT structure can incorporate water without any volume change. This was observed by SEM images of different prepared samples (Figure 4).

In Figure 4, SEM images with the same magnification are shown. It was observed that NC-N (A), NC-NT (B) and NC-C (C) gels shows a homogeneous porous structure. In contrast, NC-CT (D) shows large macro-structures and sometimes smaller sub-structures within pores. At a smaller magnification the larger pores of NC-CT gels can be seen more easily (Figure 4E). Here, macropores in the range of several micrometers were observed. It was assumed that the undefined shape of the pores was a result of a collapsed structure. If the water was evaporated due to the freeze-drying process the network structure could not stabilize the shape against gravity. As results the walls of the pores folds and buckles and ends up in undefined shape.

**Figure 5:**
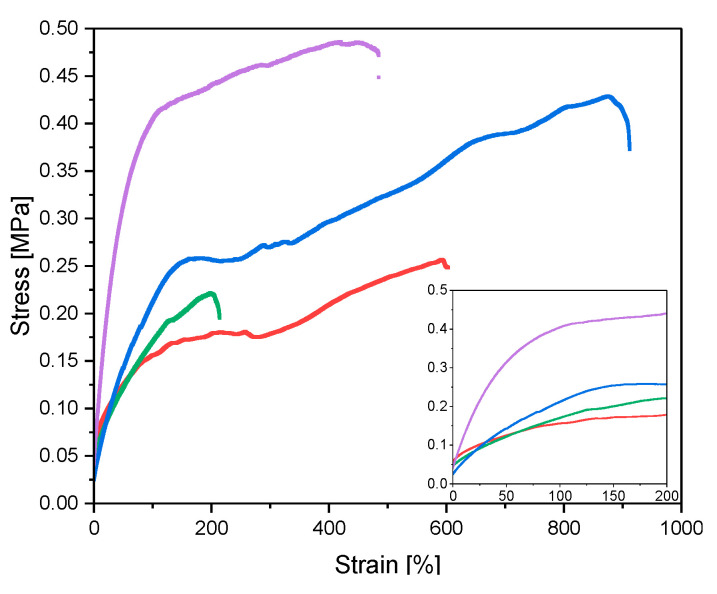
Stress strain curves of prepared NC-N (red curve), NC-NT (blue curve), NC-C (green curve) and NC-CT (purple curve) gels. Bottom right corner magnification of curves over 200% strain. All samples were swollen to 300 wt%.

To measure the tensile strength the swollen samples were elongated until they break (Figure 5). In the beginning of the experiment the gels were fixed with clamps in a predefined distance. Then the gels were prestretched with a force of 0.25 N to ensure that the measurement starts in an elongated state of the samples. These experiments were done in swollen state with a water content of 300 wt% to ensure that the differences can merely be related the network structure derived from the preparation methods. From the linear range of the stress-strain curves the E modulus was calculated (Figure 5). The values are summarized in Table 2. It was observed that the new NC-CT gels had the highest E modulus of all prepared samples.

The TEOS modified gels were stronger than the unmodified counterparts (Table 2). For NC-N and NC-NT gels it was known that the tensile strength would be increased [7,13]. This behavior was now observed for cryo gels, too. Due to silica post modification the gel stiffness was 1.3 times higher than for NC-N gels, while the stiffness of silica modified cryo gels were 2.5 times higher.

**Figure 6:**
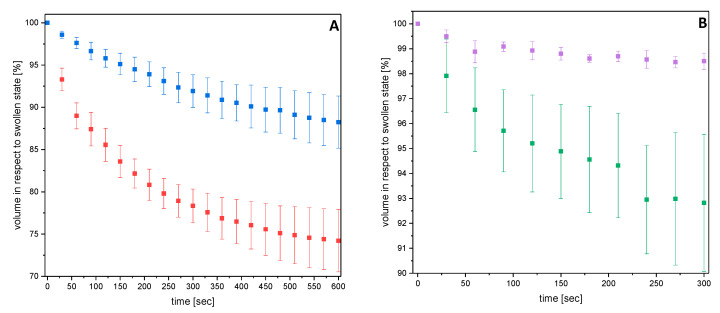
Time depending volume decrease of fully swollen NC-N (red)/NC-NT (blue) (**A**) and NC-C (green)/NC-CT (purple) (**B**) gels dropped into a water bath of 40 °C. Error bars describe standard deviation of minimum 3 samples.

To enable applications of clay gels, it is important to know the kinetic of thermo responsive behavior. Therefore, the swelling degree was analyzed by capturing time depending images of fully swollen gels put into a water bath of 40 °C. While determining changes of hydrogels length and diameter, the percent volume decrease can be calculated (Figure 6). For applications, it is important to know the initial behavior of the gels. Hence, only the initial stage of the deswelling was analyzed. It was observed that the percent volume decrease correlates with determined degree of swelling. The prepared NC-NT and NC-CT gels undergo a smaller change in degree of swelling as the corresponding NC-N and NC-C gels above the CPTT, which could be expected from Figure 2. Figure 6 shows the time which is needed that the gels undergo volume change above CPTT. This response to the external stimulus is called the response time of the sample. The response time of cryo gels was only half of NC-N gels. This behavior is in accordance to literature reports and was observed for nanocomposite gels [23]. As well, it was observed that the TEOS post modification of gels causes an almost linear dependence of volume decrease and time. This effect was observed by Haraguchi et al. for nanocomposite gels in dependence of clay contents as well [9]. Following this observation, a short linear decrease was determined for NC-CT gels, too.

## 3. Conclusions

Here a new modification of nanocomposite gel preparation by temperature treatment and TEOS modification was presented. The influence on gel properties of this route was analyzed. It was observed that the polymer cryo-clay-silica modification combines and intensifies the influence on degree of swelling, thermo responsive behavior and response time like the single modified counterpart. The performed analysis shows that the effect on increasing accepted tensile strength due to silica post modification was stronger for cryo-clay gels as for other clay gels.

## 4. Materials and Methods 

### 4.1. Chemicals

For polymer gel synthesis, *N*-isopropylacrylamide (NIPAAm) (98 %) was purchased from TCI, potassium persulfate (KPS) (99 %) from Merck (Darmstadt, Germany) and *N,N,N’,N’*-tetramethyl ethylene diamine (TMEDA) (99 %) from Fluka (Seelze, Germany) and synthetic hectorite clay Laponite® XLS (92.32 wt% of Mg_5.34_Li_0.66_Si_8_O_20_(OH)_4_Na_0.66_ and 7.68 wt% of Na_4_P_2_O_7_) from Rockwood Ltd. (Newry, UK). For silica post modification tetraethyl orthosilicate (TEOS) (98 %) was purchased from Sigma-Aldrich (Taufkirchen, Germany). Ethanol and hydrogen chloride (37 %) were used in technical grade.

### 4.2. General Procedure for NC-gel and NC-cryogel Preparation

The preparation of NIPAAm gels was similar to the procedure described in the literature [11,23]. For preparation of NC-10 gel the ratio of NIPAAm and clay (Laponite® XLS) was set to 10 wt% relative to the amount of water. Initially, clay (1 g) was stirred in deionized water (9 mL) for 10 min to get a homogenous mixture. Afterwards, NIPAAm (1 g) was added to the mixture and stirred for additional 10 min. The mixture was flushed with argon for at least 10 min while it was cooled by an ice/water bath. The polymerization was started by adding 1 mL KPS solution (0.3 mg/mL) in water and TMEDA (24 µL) to the cold monomer mixture. The mixture was transferred quickly to cylindric frames to form polymer gels. For preparation of NC-gel, the filled frames were stored at room temperature for a minimum of 24 h, while frames for NC-cryogel preparation were stored at −20 °C for 48 h. After the polymerization the samples were stored in a water bath for lixiviation of unreacted monomers. The samples were stored in water until further use but at least overnight. The successful application of clay within the gels was analyzed by TGA. For NC-N gels, a clay content of 40 wt% and for NC-C gels of 39 wt% was observed.

### 4.3. Silica Post Modification of NC-gel and NC-cryogels

The synthesized NC-gels and NC-cryogels were post modified with silica in analogy to literature [13]. The prepared gels were swollen at room temperature in ethanol for 5 h, while ethanol was exchanged every hour. Afterwards, the ethanol was replaced by TEOS in ethanol (1 M) solution for 30 min. To perform polycondensation the former solution was removed, and the gels were covered by an ethanol/water/hydrogen chloride (58 mL/8.5 mL/2.5 µL) solution for 10 min. The solution was removed, and the gels were stored for 12 h at 50 °C. The gels were washed with water several hours and dried at ambient conditions.

Degree of TEOS modification was measured with TGA and was determined for modification of NC-gels to 7 wt% and for NC-cryogels to 10 wt%.

### 4.4. Thermo-Responsive Behaviour

The degree of swelling was calculated by determining the diameter of cylindric samples via a microscope (Stativ AD purchased from Hund/Wetzlar, Wetzlar, Germany) with a camera from IDS and a microscopic ruler. With that the volume of the samples were calculated. For temperature depending swelling, the gels were immersed in a water chamber with temperature control and equilibrated for 1 h. For capture thermo-responsive behavior the chamber was heated to a certain temperature and the diameter of the gels was measured. 

For swelling kinetic determination at room temperature equilibrated gels were immersed in a water bath of 50 °C. Immediately after immersing, images of the gels were taken every 30 s to observe volume decrease.

For degree of swelling determined by scaling, the samples were stored in a closed vessel and equilibrated for 1 h. Afterwards, the samples were taken out, the surface was dried with a cloth and the samples were scaled.

The degree of swelling was calculated in respect to dry volume or mass. This state was reached by drying the samples at 50 °C over several days until a constant mass was reached.

### 4.5. Tensile Strength Measurements

For characterizing tensile properties, samples were dried completely at 50 °C for 72 h. To adjust the same degree of swelling for all samples, certain amounts of water (300 wt% relative to dry weight of the samples) were added. Before tensile measurements, the diameter of the gels was measured separately. The tensile strength was measured with a Zwick/Roell-1446 universal testing machine (Ulm, Germany). After a preforce was reached, the gels were stretched with a test speed of 50 mm/min until the gel broke. The gels were cylindrical with an entire length of 5–7 cm, while clamping distance was 15 mm.

### 4.6. Sample Preparation for SEM

After lixiviation, the prepared NC gels were swollen in water overnight. Afterwards, the swollen samples were frozen with liquid nitrogen and freeze-dried overnight. Next, the samples were cut into smaller pieces. To image the inner structure the gel samples were on one end attached to an SEM sample holder. For imaging, a layer of 5 nm Au/Pd (ratio: 80/20) was sputtered onto the surface. The samples were analyzed with a NEON^®^ 40 microscope purchased from Zeiss.

## Figures and Tables

**Table 1:** Average volume degree of swelling of each polymer-clay composite; bracketed values describe standard deviation of a minimum of 3 samples.

Sample Type	NC-N	NC-NT	NC-C	NC-CT
volume degree of swelling [-] at 25 °C	7.74 (± 1.22)	2.92 (± 0.41)	6.81 (± 0.85)	1.09 (± 0.07)

**Table 2:** Calculated E modulus of different gels swollen to a water content of 300 wt%. Values in brackets describe standard deviation of at least 6 separately prepared samples. E modulus is calculated over first 50 % strain.

Sample Type	NC-N ^a^	NC-NT	NC-C	NC-CT
E modulus [kPa} 300 wt% swelling	1.63 (± 0.37)	2.30 (± 0.78)	1.85 (± 0.38)	3.69 (± 0.96)
stress at break [MPa] 300 wt% swollen	0.376 (± 0.11)	0.471 (± 0.10)	0.178 (± 0.03)	0.457 (± 0.06)

^a^ Preforce was set to 0.5 N.
